# Effect of shade level and mulch type on growth, yield and essential oil composition of damask rose (*Rosa damascena* Mill.) under mid hill conditions of Western Himalayas

**DOI:** 10.1371/journal.pone.0214672

**Published:** 2019-04-04

**Authors:** Meenakshi Thakur, Vinod Bhatt, Rakesh Kumar

**Affiliations:** 1 Academy of Scientific and Innovative Research, New Delhi, India; 2 Agrotechnology of Medicinal, Aromatic and Commercially Important Plants Division, CSIR-Institute of Himalayan Bioresource Technology, Palampur (HP), India; 3 Natural Product Chemistry and Process Development Division, CSIR-Institute of Himalayan Bioresource Technology, (Council of Scientific and Industrial Research), Palampur (HP), India; University of Vigo, SPAIN

## Abstract

A field experiment was conducted at the experimental farm of CSIR-Institute of Himalayan Bioresource Technology, Palampur, India for two consecutive years (2015–16 and 2016–17). The aim of the study was to test the hypothesis whether different shade level and mulch type would influence the growth, flower yield and essential oil profile of *R*. *damascena*. Yield attributes *viz*., numbers of flowers plant^-1^, fresh flower weight plant^-1^, flower yield, and essential oil yield were significantly higher under open sunny conditions as compared to 25% and 50% shade levels. However, plants grown under 50% shade level recorded significantly higher plant height (cm), plant spread (cm) and the lowest numbers of branches as compared to control. Among mulches, black polyethylene mulch recorded significantly higher growth, and yield attributes of damask rose as compared to other mulches. Black polyethylene mulch recorded 74.5 and 39.2% higher fresh flower yield as compared to without mulch, during 2015–16 and 2016–17, respectively. Correlation studies showed a positively significant correlation between quality and quantity traits. A total of twenty-six essential oil compounds were identified which accounted for a total of 88.8 to 95.3%. Plants grown under open sunny conditions along with the applications of black polyethylene mulch produced a higher concentration of citronellol and *trans*-geraniol. Damask rose planted in open sunny conditions and mulched with black polyethylene sheet recorded significantly higher flower yield.

## 1. Introduction

Damask rose (*Rosa damascena* Mill.) is considered as one of the important aromatic crops. The plant is highly valuable as it has application in perfumery, cosmetics and flavors industries. Besides this, it has application in pharmacology such as anti-HIV, antibacterial, antioxidant, hypnotic, antidiabetic activities [1] and is used to cure depression, insomnia and for stress reduction [2]. Bulgaria, Turkey, Morocco, Iran, India, Egypt, China, and Russia are known to produce damask rose for producing rose oil, rose water, concrete, attar and absolute [3]. The price of its essential oil varies from 7 to 10 lakh kg^-1^ [2]. The quality of essential oil of damask rose is due to the high percentage of the monoterpene alcohols such as citronellol, *trans*-geraniol, linalool, phenyl ethyl alcohol and hydrocarbons such as nonadecene and nonadecane. The growth and yield of damask rose are affected by agronomic factors *viz*., nutrient management, varieties, harvesting stage, distillation process, diurnal variability and storage conditions of flowers.

Light is one of the most important environmental factors for plant growth and development [4]. Different light intensities influence plant growth, leaf gas exchange, and water use efficiency. In aromatic crops, different light intensities have been demonstrated to alter essential oil content and composition. The substantial amount of light energy is required to reduce carbon which combines with CO_2_ producing oxygen, carbohydrates, ATP and NADPH. The sugars converted into amino acids, hormones, and secondary metabolites. However, plants grown under shade level leads to reduce the stomatal conductance, photosynthesis rate, ATP synthesis, carbon assimilation and plant growth [5,6]. The study of microclimate modification by using different green shade nets found that different crops behaved differently under shade conditions [7]. These variations in the microclimate modify the rate of CO_2_ assimilation and photosynthesis, consequently, crop growth and productivity. Crop yield and productivity can also be increased by application of mulch in the soil as it improves soil conditions for plant growth, including soil temperature, soil moisture, weed control, reduction in leaching of fertilizers [8]. Mulching is also known to buffer soil temperature, increases early production and higher yields along with better quality; enhance water and fertilizer use efficiency and decrease the incidence of pests [9]. Therefore, different types of mulching materials are used to change the micro environment along with the improvement of crop growth and yield.

Plant acclimation to different light intensities depends on environmental conditions and plant genotype, and thus is species-specific [10,11]. There is a dearth of information on shade effect and mulch type for the production of damask rose in the world. Modification of microclimate by shade and mulch is an alternative approach to meet the increasing demand of damask rose under such conditions. However, the effects of shade level and mulch type on *R*. *damascena* have not been studied under Western Himalayan conditions. In view of the above facts, an experiment was conducted to study the effect of shade level and mulch type on growth, flower yield, essential oil content, and composition of damask rose under mid hill conditions of Western Himalayas.

## 2. Materials and methods

### 2.1 Experimental site

**Fig 1:**
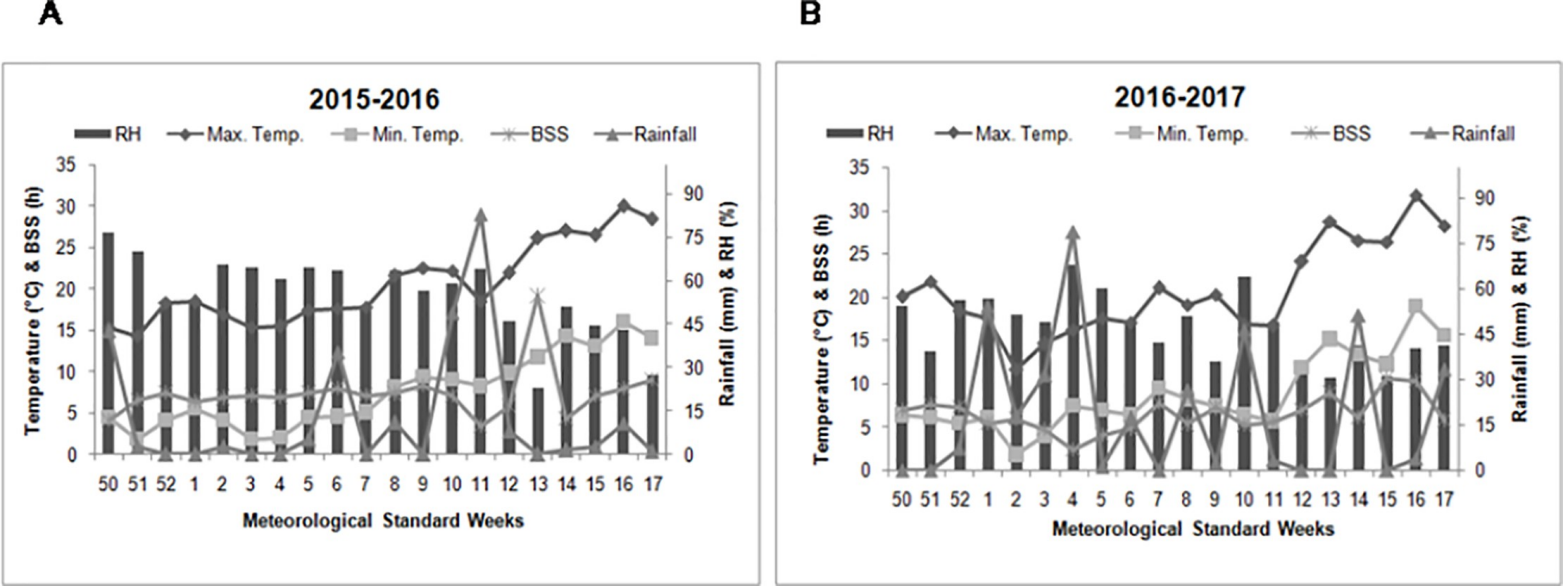
**Meteorological standard weeks (A) during growing season of damask rose (2015–2016) (B) during growing season of damask rose (2016–2017)**.

The field experiment was conducted at the experimental farm of CSIR-Institute of Himalayan Bioresource Technology, Palampur, India, located at 32°06’05”N latitude, 76°34’10”E longitude and an altitude of 1390 m above mean sea level during 2015–16 and 2016–17. Maximum temperature (14.3 to 30.0°C and 11.6 to 31.9°C), minimum temperature (1.8 to 16.0°C and 1.7 to 19.0°C), relative humidity (22 to 76% and 31 to 68%), and total rainfall 254.8 mm and 373.2 mm) were recorded during both the seasons (Fig 1).

### 2.2 Experimental details

Damask rose variety Jwala was used to study the effect of microclimate modification during 2015–16 to 2016–17. The soil of the experimental field was silty clay in texture (10.76% sand, 55.48% silt and 33.76% clay), acidic in reaction (pH 5.25), low in organic carbon (0.2%) and exhibited a low availability of nitrogen (49.6 mg/kg of soil), low phosphorous (3.1 mg/kg of soil) and high potassium (182.2 mg/kg soil). Application of nutrients NPK was applied in the ratio of 120:60:40 kg/ha through fertilizer urea (46% N), single super phosphate (16% P_2_O_5_) and muriate of potash (60% K_2_O), respectively. The experiment was laid out in a split plot design (SPD) with four replications. The agro-shade net houses (shade level) were organized in main plots, while the different mulches were subjected to sub-plot. Shade houses (3 m height x 12 m width x 22 m length) were constructed on a 0.2 ha section of an open field at the Chandpur farm of the institute. Green polypropylene shade fabrics of different mesh gauges were erected on frames to simulate shade treatments: 25 and 50%. Frames without shade fabric served as control (i.e., 0% shade). The shade houses were placed on ground cover in the open field, 2 m a part to prevent treatment overlap.

The experimental area was divided into three blocks to account for any shading effect from adjacent sides. After pruning of damask rose bushes in the month of December, nine treatments consisting of three shade net houses *viz*., control (without shade), 25% shade and 50% shade level were made. Three mulch types *viz*., control (without mulch), organic mulch at the rate of 5 t/ha (poplar leaf mulch) and black polyethylene mulch (25/30 micron size) in sub plots were organized.

### 2.3 Growth and yield attributes

Biometric observations were recorded at the time of flowering. Growth information on plant height (cm), numbers of branches plant^-1^, plant spread (cm) and leaf area index (LAI) was recorded. Five plants plot^–1^ were selected for recording plant height, plant spread, number of branches plant^–1^, and leaf area. Plant height was measured from the ground level to tip of the top leaf. Plant spread was recorded in North-South and East-West directions. Yield parameters *viz*., numbers of flowers plant^-1^, fresh flower weight plant^-1^ (g) flower yield (kg ha^-1^), essential oil yield (kg ha^-1^), essential oil content (%) and essential oil components were recorded. Plant height and leaf area index (LAI) was measured with the help of measuring scale and digital plant canopy imager CI-110/120, respectively. Photosynthetically Active Radiations (PAR) under each shade and mulch treatment was measured during the flowering season with a LI-190 quantum sensor (LI-COR, Inc., USA). The observations were taken around 8:00 AM to 4:00 PM after each interval of two hours as the sun’s angle to normal (zenith angle) is less than 2°C on most days of observations. Each reading was expressed as μmol^-1^m^-2^s^-1^.

### 2.4 Extraction of essential oil

Damask rose flowers were collected separately from each treatment during April and mid of May months of the crop season. In each treatment, 1000 g flowers were used for distillation. Damask rose flowers were harvested early in the morning (5:00–7:00 AM) by manual plucking and were immediately hydro-distilled in the Clevenger type apparatus within 10 minutes after harvest to prevent the loss of volatile compounds from the flowers. The ratio of flower and water was 1:2 (w/v) and the essential oil content of damask rose was measured as mL and % ratio (v/w) on the fresh weight basis. Essential oil yield (kg ha^-1^) was calculated by multiplying fresh flower yield, essential oil content and specific gravity (0.85) of rose essential oil. The extracted essential oil samples were dried over anhydrous sodium sulphate and were stored at 4°C before analysis. Three replicates were used to identify the essential oil composition through GC and GC-MS.

### 2.5 Compounds identification by GC-MS and GC

Gas chromatography—Mass spectrometry (GC-MS) analysis were carried out on a Shimadzu QP 2010 GC-MS system equipped with AOC-5000 auto-injector and DB-5 capillary column (30 m × 0.25 mm, 0.25 mm film thickness) from SGE, Australia; in which helium at the rate of 1.28 mL min^-1^, sample injection of 2 μL with a split ratio of 10; ionization energy of 70 eV was used. Ion source temperature of 200°C, injector temperature of 250°C was maintained. The oven temperature used at the initial stage was 40 ^o^C for 4 Min, at rising 4°C min^-1^ to 220°C and then held isothermal at 220°C for 4 min.

The composition of rose oil samples was evaluated by a Shimadzu GC-2010 gas chromatograph (Shimadzu, Tokyo, Japan) equipped with flame ionization detector (FID) and DB-5 (J&W Scientific, Folsom, USA) fused silica capillary column (30 m × 0.25 mm, i.d.;0.25 μm film thickness). Nitrogen was used as carrier gas at the column with the velocity of 1.24 mL min^-1^ flow rate. The oven temperature was programmed at 40°C for 4 min and then increased at the rate of 4°C min^-1^ from 40–220°C. The temperature of injector and detector was maintained at 250°C and samples were injected in split mode. The compounds were identified based on retention indices and peak area percentage of the chromatogram. Retention indices (RI) were calculated by using homologous series of n-alkanes (C8–C24), and then compounds were identified by comparing R.I values, mass spectra, and library databases of NIST-MS (National Institute of Standards and Technology-mass spectral) database [12,13].

### 2.6 Statistical analysis

Statistical analysis of data was done according to the standard analysis of variance (ANOVA) by using the software SYSTAT-12 (SYSTAT Software Inc., Chicago, IL, USA). Variations among treatments were assessed by LSD values at 5% probability (p≤ 0.05) level were processed by multiplying standard error of the difference. Correlation studies were carried out to investigate the relationship between growth and yield parameters. While, the standard deviation method was used to prepare essential oil compounds using excel stats software. Multivariate principal component analysis (software PAST3) was used to evaluate the effect of treatment combinations of shade level and mulch type on essential oil compounds.

## 3. Results and discussion

### 3.1 Photosynthetic active radiation

**Fig 2:**
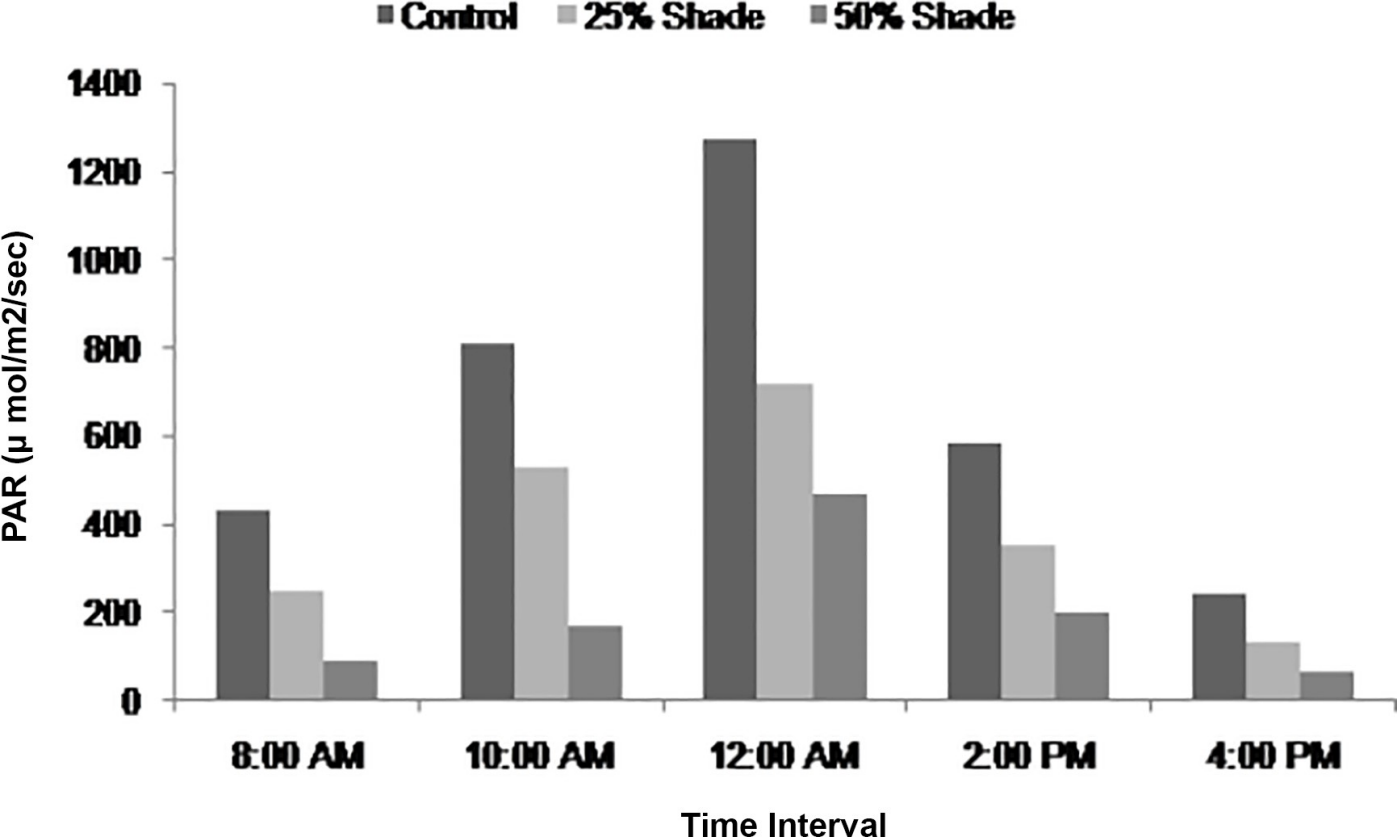
Photosynthetic Active Radiation (PAR) at different time intervals during flowering season of damask rose.

Plant tolerance to high or low PAR condition is regulated by the microclimate modification. Increase in shade level reduces PAR and alters light intensity, affecting the photosynthetic rate, and crop yield production. Higher PAR was recorded at mid day under control (1500 μ mol^-1^m^-2^s^-1^) followed by 25% (800 μ mol^-1^m^-2^s^-1^) and 50% shade level (500 μ mol^-1^m^-2^s^-1^) (Fig 2). The results were similar to the studies of clary sage (*Salvia sclarea*), and behavior of rose cultivar in different shade levels in which decreased PAR under increased shaded conditions was reported [4,5].

### 3.2 Effect of shade level on growth parameters

Microclimate modification significantly influenced crop growth and yield of damask rose. The plants grown under shade conditions were taller than those grown under open sunny conditions. The growth parameters were significantly affected by different shade levels during both the years (Table 1). Plant height was significantly higher under 50% shade (137.4 and 144.7 cm), as compared to 25% shade (125.2 and 127.4 cm), and control (100.3 and 119.7 cm), during both the years. This may be due to the fact that plant grown in shade resembled etiolated plants, which have an unwanted tall appearance and are more apical dominant. It also produces a phototropism response to modify plant growth and development to receive enough light [14]. Results were similar to the studies of rose scented geranium (*Pelargonium graveolens* family; Geraniaceae) and bottle gourd (*Lagenaria vulgaris*) in which growth parameters were higher in shaded conditions [15,16]. Numbers of branches were 31.4 and 22.8% higher in open sunny conditions as compared to 50% shade level during 2015–2016 and 2016–2017, respectively. This may be due to the absorption of lesser PAR under shade conditions that have reduced the photosynthetic and respiration rate that usually inhibits plant growth and productivity by affecting the gas exchange as compared to full sunlight [17]. During both the years of studies, plant spread (N-S) was significantly higher in 50% shade level as compared to open sunny conditions (control). Significantly, lowest plant spread was recorded under open sunny conditions which might be due to higher absorption of PAR, that had enhanced the photosynthetic rate compared to shaded conditions (Fig 2). The presented data revealed significantly higher LAI under 50% shade level followed by 25% shade and control, respectively, for both years of studies. Similar results were observed by modifying the microclimate in *S*. *officinalis* to different shade levels resulted in maximum leaf areas when planted in 50% shade [5,18,19].

**Table 1:** Effect of shade level and mulch type on growth parameters of damask rose.

Treatment	Plant height (cm)		Numbers of branches plant^-1^		Plant spread (cm)				Leaf area index (LAI)	
					N-S		E-W			
	2015–2016	2016–2017	2015–2016	2016–2017	2015–2016	2016–2017	2015–2016	2016–2017	2015–2016	2016–2017
Shade level										
Control (0% Shade)	100.3^c^	119.7^c^	22.2a	25.3^a^	93.5^c^	99.7^c^	111.2^c^	116.3	4.0^bc^	4.8^c^
25% Shade	125.2^b^	127.4^b^	18.1^b^	23.8^ab^	106.9^b^	112.3^ab^	127.9^b^	132.6	5.0^ab^	6.3^ab^
50% Shade	137.4^a^	144.7^a^	16.9^bc^	20.6^c^	122.6^a^	118.9^a^	140.4^a^	141.6	5.6^a^	6.6^a^
SEm±	2.7	1.1	0.5	0.7	0.9	2.8	1.6	3.5	0.3	0.3
LSD (p ≤ 0.05)	9.6	3.7	1.7	2.4	3.2	9.5	5.5	NS	1.1	0.9
Mulch type										
Without Mulch	109.0^c^	121.4^c^	17.9	21.4^bc^	104.2^b^	96.8^c^	122.2^bc^	112.2^c^	4.8	5.5
Organic Mulch	123.1^ab^	131.4^b^	19.3	23.7^ab^	117.2^a^	113.2^b^	133.7^a^	131.6^b^	4.9	5.8
Black polyethylene Mulch	129.9^a^	139.1^a^	20.0	24.6^a^	101.5^bc^	120.9^a^	123.5^b^	146.7^a^	4.8	6.4
SEm±	3.0	1.5	0.9	0.8	2.6	3.2	2.8	10.0	0.2	0.2
LSD (p ≤ 0.05)	8.9	4.5	NS	2.5	7.6	9.4	8.2	11.2	NS	NS

Note: N-S: North South; E-W: East West; NS: Not significant; LSD: Least Significant Difference; SEm: Standard Error of Mean;LAI: Leaf area index; Mean values with same letter in same columns do not differ significantly (p≤0.05)

### 3.3 Effect of different mulch types on growth parameters

Growth parameters were significantly affected by different mulch (Table 1). During both the years, plant height was significantly higher under black polyethylene mulch (129.9and 139.1 cm), as compared to control. Results were in accordance with the studies of sweet-scented geranium and stevia in which plant height increased in mulched conditions as compared to control [20,21]. This is due to increased temperature under black polyethylene mulch that has enhanced the microbial activity of nitrifying bacteria that access the nitrification and nitrate to plant roots under soil [22,23]. Significantly higher numbers of branches were recorded under black polyethylene mulch followed by organic and without mulch (control) during both the years. During the first year, organic mulch recorded significantly higher plant spread (N-S and E-W direction) followed by black polyethylene mulch and control. However, during the second year, plant spread in both directions was significantly higher in black polyethylene mulch followed by organic mulch and control. Similar results were reported in the studies of African marigold in which maximum plant spread was recorded in plastic mulch [24]. Plant growth is influenced by the use of mulch, such as moisture content, reduced competition for light and availability of nutrients, absence of weeds, pest reduction and increased soil temperature [24]. LAI was not affected significantly by different mulch during both the years.

### 3.4 Effect of shade levels on yield attributes

Yield attributes of damask rose were significantly affected by shade level and mulch types (Table 2). Significantly higher numbers of flowers were recorded under open sunny conditions as compared to 25 and 50% shade level during both the years. Light affected chlorophyll content, photosynthetic enzyme activity, stomatal opening, and the distribution of carbohydrates in plants [25,26,27]. A perusal of data presented in Table 2 revealed that fresh flower weight plant^-1^ was significantly affected by shade level and mulch type. At 50% shade level, fresh flower weight plant^-1^ was found significantly lowest (105.3 and 207.2 g), however, control recorded significantly higher fresh flower weight plant^-1^ (511.3 and 381.6 g), respectively, during both the years. During the second year of study, fresh flower weight per plant was found at par with control and 25% shade level. It may be because of the lesser weed growth, more numbers of branches and numbers of flowers resulted in higher fresh flower weight under open sunny conditions and 25% shade level. Similar results were observed in clary sage in which fresh weight biomass was reduced with increase in shade level [28]. Reduction in light intensity reduces the rate of photosynthesis and rate of the growth. While, more vegetative growth, lesser numbers of flowers resulted in significantly lesser fresh flower weight plant^-1^ under 50% shade conditions [29,30].

**Table 2:** Effects of shade level and mulch type on yield attributes and yield of damask rose.

Treatment	Number of flowers plant^-1^		Fresh flower weight plant^-1^ (g)		Flower yield (kg ha^-1^) Essential oil yield (kg ha^-1^)			
	2015–2016	2016–2017	2015–2016	2016–2017	2015–2016	2016–2017	2015–2016	2016–2017
Shade level								
Control (0% Shade)	186.7^a^	131.8^a^	511.3^a^	381.6^a^	4544.7^a^	3392.4^a^	2.0^a^	1.5^a^
25% Shade	79.2^b^	111.5^b^	230.7^b^	334.2^ab^	2050.3^b^	2970.3^ab^	0.9^b^	1.4^ab^
50% Shade	34.2^c^	78.7^c^	105.3^c^	207.2^c^	936.1^c^	1841.4^c^	0.4^c^	0.9^c^
SEm±	5.4	7.2	19.6	44.1	174.1	392.4	0.01	0.08
LSD (p ≤ 0.05) 6.2		8.3	22.6	50.9	200.9	452.7	0.05	0.3
Mulch type								
Without Mulch	72.8^c^	90.1^c^	203.1^c^	256.3^c^	1805.0^c^	2278.1^c^	0.8^c^	1.0^c^
Organic Mulch	102.1^b^	109.0^b^	289.9^b^	309.7^b^	2576.7^b^	2753.2^b^	1.2^ab^	1.3^b^
Black polyethylene Mulch	125.2^a^	122.9^a^	354.3^a^	356.9^a^	3149.4^a^	3172.7^a^	1.4^a^	1.5^a^
SEm±	11.2	6.5	30.9	44.1	274.3	147.0	0.3	0.2
LSD (p ≤ 0.05)	11.0	6.5	30.6	16.4	271.5	145.5	0.1	0.1
LSD: Interaction (S X M)	19.1	11.2	52.9	28.4	470.3	252.0	0.2	NS

Note: S: Shade; M: Mulch; LSD: Least Significant Difference; SEm: Standard Error of Mean; Mean values with same letter in same columns do not differ significantly (p≤0.05)

Plants grown under open sunny conditions produced significantly higher flower yield during both the years (Table 2). During the first year, open sunny conditions recorded significantly higher flower yield (4544.7 kg ha^-1^) than 25% (2050.3 kg ha^-1^) and 50% shade level (936.1 kg ha^-1^), respectively. During the second year, plants under open sunny conditions registered significantly higher flower yield as compared to 50% shade level but remained at par with 25% shade level. It may be attributed to the favorable environment for increased numbers of branches consequently increased the numbers of flowers that resulted into higher flower yield. The essential oil obtained by hydro distillation in Clevenger-type apparatus was influenced significantly by different shade levels and mulch types (Table 2). Significantly higher essential oil yield was recorded in open sunny conditions as compared to 25% and 50% shade level during the first year of study. Similarly, during the second year, essential oil yield was significantly higher in control as compared to 50% shade level but remained at par with 25% shade level. This may be due to the higher flower yield and essential oil content under control and 25% shaded conditions as compared to 50% shade level (Table 2). The production and quality of essential oil of medicinal and aromatic species responds differently to shade conditions due to genetic makeup and environmental conditions. Essential oil yield of *Thymus vulgaris* and *Matricaria chamomilla* increased under open sunny conditions, however, *Ocimum gratissimum*, *Anethum graveolens* and *Pothomorphe umbellate* recorded significantly higher essential oil yield when cultivated under shade [31].

### 3.5 Effect of mulch type on yield attributes

Among mulch types, black polyethylene mulch recorded significantly higher number of flowers as compared to organic mulch and control during both the years. Black polyethylene mulch, registered 72.0 and 36.4% higher number of flowers as compared to control, during 2015–16 and 2016–17, respectively. It might be due to favorable soil temperature and a moisture condition that has enhanced the microbial activity in the soil resulting in enhanced nutrient availability and physiological activities of plant which ultimately resulted in the higher number of flowers [24,32]. Black polyethylene mulch produced a significantly higher fresh flower weight plant^-1^ as compared to organic mulch and control. Black polyethylene mulch, enhanced the fresh flower weight plant^-1^ by 74.4 and 39.3% as compared to control during both the years. Similar results were reported under black polyethylene mulch in rose [8]. Black polyethylene mulch, retained higher soil moisture content with efficient use of nutrients, which might have maximized the number of flowers, hence, fresh flower weight.

Plants mulched with black polyethylene, produced significantly higher flower yield (3149.2 and 3172.7 kg ha^-1^) as compared to organic mulch (2576.7 and 2753.2 kg ha^-1^) and without mulch (1805.0 and 2278.1 kg ha^-1^), respectively, during both the years. Black polyethylene mulch, recorded an increase of 74.5 and 39.3% as compared to without mulch, respectively. Mulch types, could be attributed to water use efficiency, alter the soil temperature and soil moisture content, proper utilization of nutrients which might favor the environmental conditions for the plant. The results obtained were consistent with the studies of *Pogostemon cablin* and *Java citronella* [32,33]. Among mulches, black polyethylene mulch recorded significantly higher essential oil yield as compared to organic and control during both the years.

#### 3.5.1 Interaction effect on yield attributes and yield

Interaction effect of shade level and mulch type on numbers of flowers plant^-1^ and fresh flower weight plant^-1^ were found significant during both the years (Table 2). Plants grown under open sunny conditions recorded significantly higher numbers of flowers plant^-1^ as compared to 25 and 50% shade level at each mulch type. Black polyethylene mulch recorded significantly higher number of flowers plant^-1^ and fresh flower weight plant^-1^ as compared to organic mulch and control at each shade level. In all treatment combinations, black polyethylene mulch under control produced significantly higher number of flowers plant^-1^ and fresh flower weight plant^-1^. Interaction effect of shade and mulch factors on fresh flower yield of damask rose was found significant during both the years. Open sunny conditions produced significantly higher flower yield, however, shaded condition of 50% level produced significantly lower flower yield. Plants under open sunny conditions produced significantly higher flower yield with black polyethylene mulch. The different mulch produced significant difference with different light intensities. Black polyethylene mulch, produced a significantly higher flower yield followed by organic and without mulch, respectively in open sunny conditions.

### 3.6 Correlation analysis

The correlation study for phenotypic level, growth and yield attributes are given in Table 3. Numbers of flowers plant^-1^ and fresh flower weight plant^-1^ produced significantly (p ≤ 0.01) higher positive correlation with flower yield (0.99), essential oil content (0.77), while, showed positive correlation with numbers of branches plant^-1^ (0.44). Similar results were observed in correlation matrix of damask rose in which data revealed that flower yield was significantly and positively correlated with the number of flower [34]. Plant height (cm) had highly positive significant correlation for LAI (0.89), N-S plant spread (0.88) and E-W plant spread (0.82), however, had positive correlation for number of branches plant^-1^. Numbers of branches plant^-1^ produced significant positive correlation for essential oil content (0.71), while, showed positive correlation for growth and yield attributes. N-S plant spread showed highly significant positive correlation for E-W plant spread (0.94) and showed significant and positive correlation for LAI (0.78). Likewise, E-W plant spread showed positive correlation for LAI (0.78). Essential oil content produced significantly higher positive correlation for flower yield (0.77). Similar results were observed in *Tanacetum parthenium*, in which positive correlation was observed between essential oil % and flower yield [35].

**Table 3:** Estimate of correlation coefficients at the phenotypic levels for quantitative and quality traits during crop season (Pooled data of two years).

Parameters	Fresh flower weightplant^-1^ (g)	Plant height (cm)	Number of branches plant^-1^	N-S plant spread (cm)	E-W plant spread (cm)	Leaf area index (LAI)	Essential oil content (v/w%)	Flower yield (kg ha^-1^)
Numbers of flowers plant^-1^	0.99**	-0.69	0.44	-0.59	-0.53	-0.77	0.77*	0.99**
Fresh flower weight plant^-1^ (g)		-0.67	0.45	-0.59	-0.51	-0.76	0.77*	0.99**
Plant height (cm)			0.14	0.88**	0.82**	0.89**	-0.24	-0.67
Numbers of branches plant^-1^				0.24	0.32	0.20	0.71*	0.45
N-S Plant spread (cm)					0.94**	0.78*	-0.08	-0.59
E-W plant spread (cm)						0.78*	-0.10	-0.51
Leaf area index (LAI)							-0.33	-0.76
Essential oil content (v/w%)								0.77*

Note

*, **, Significant correlation with p values ≤ 0.05, ≤ 0.01, respectively.

### 3.7 Essential oil yield and composition

Essential oil yield ranged from 0.051 to 0.055% in different treatments (Table 4). Damask rose essential oil yield was not significantly affected by different shade level and mulch types. The analysis of essential oil of *R*. *damascena* led to the identification of twenty six compounds. The total area percentages of damask rose components were 88.8 to 95.3%. The components were grouped into oxygenated monoterpene (53.2 to 68.0%), oxygenated sesquiterpene (3.6 to 6.3%), sesquiterpene hydrocarbon (3.0 to 4.9%) and aliphatic hydrocarbon (16.1 to 31.8%) (Table 4). The analyzed component of damask rose essential oils mainly contains oxygenated monoterpenes and aliphatic hydrocarbon as a major fraction. The major components were citronellol (35.5 to 49.2%), *trans*-geraniol (12.6 to 18.4%), nonadecane (8.9 to 15.1%), heneicosane (3.8 to 8.7%) and nonadecene (1.6 to 3.6%).

**Table 4:** Effect of shade level and mulch type on essential oil compounds of damask rose (Pooled data of two years).

				Area (%)								
	Treatment			Control			25% Shade			50% Shade		
Sr. No.	Compounds	Lit. R.I	Exp.R.I	WM	OM	PM	WM	OM	PM	WM	OM	PM
	**Oxygenated Monoterpene**											
1	Linalool	1098	1100	1.1±0.5	1.1±0.4	1.5±0.1	0.9±0.0	0.8±0.1	1.3±0.2	1.3±0.3	1.1±0.5	1.2±0.1
2	*trans*-rose oxide	1106	1115	0.6±0.1	0.6±0.2	0.8±0.1	0.6±0.0	0.5±0.2	0.6±0.1	0.6±0.1	0.5±0.4	0.7±0.0
3	Phenyl ethyl alcohol	1110	1116	0.1±0.1	0.1±0.1	0.1±0.0	0.2±0.1	0.1±0.1	0.1±0.1	0.1±0.0	0.3±0.4	0.2±0.1
4	*cis*-rose oxide	1111	1118	0.2±0.3	0.1±0.1	0.1±0.1	0.1±0.0	0.3±0.4	0.3±0.4	1.1±0.0	0.1±0.1	0.1±0.1
5	Terpineol	1189	1191	1.1±0.0	1.0±0.3	1.2±0.2	1.0±0.0	0.7±0.3	0.9±0.2	0.7±0.5	1.0±0.1	0.9±0.0
6	Citronellol	1228	1238	40.0±0.0	45.5±8.5	**49.2±1.8**	40.8±2.0	35.5±9.1	**46.4±2.6**	43.3±0.6	44.2±1.0	**46.5±3.3**
7	*trans*-geraniol	1255	1271	12.6±0.4	13.0±8.6	**14.4±4.1**	15.1±4.0	15.3±10.3	**18.4±1.2**	12.8±0.1	15.6±0.3	**15.6±3.0**
	**Sub Total**			55.7±0.2	61.4±2.6	**67.3±0.9**	58.7±0.9	53.2±0.4	**68.0±0.7**	59.9±0.2	62.8±0.4	**65.2±0.9**
	**Oxygenated Sesquiterpene**											
8	Citronellyl acetate	1354	1354	0.8±0.2	0.8±0.1	0.7±0.3	0.5±0.1	0.5±0.1	0.5±0.1	0.6±0.1	0.5±0.0	0.5±0.0
9	Eugenol	1356	1367	0.4±0.1	0.3±0.1	0.3±0.1	0.1±0.0	0.2±0.1	0.2±0.1	0.2±0.0	0.2±0.0	0.2±0.1
10	Geranyl acetate	1383	1383	2.8±0.6	2.4±1.0	2.1±0.6	1.2±0.1	1.2±0.3	1.8±0.8	2.1±1.1	1.0±0.5	1.4±0.6
11	Methyl Eugenol	1401	1401	0.9±0.3	1.0±0.2	0.8±0.1	0.8±0.1	0.7±0.1	0.6±0.0	0.9±0.1	0.8±0.2	0.8±0.1
12	Farnesol	1722	1717	1.4±0.5	1.1±0.0	0.9±0.1	1.3±0.2	1.2±0.5	0.9±0.1	1.2±0.3	1.1±0.5	1.0±0.2
	**Sub Total**			6.3±0.3	5.6±0.3	4.8±0.3	3.9±0.1	3.8±0.2	4.0±0.2	5.0±0.2	3.6±0.2	3.9±0.1
	**Sesquiterpene Hydrocarbon**											
13	Elemene	1391	1396	0.9±0.1	0.8±0.2	0.9±0.4	0.6±0.1	0.6±0.1	0.6±0.2	1.4±0.6	0.6±0.0	0.5±0.0
14	Caryophyllene	1404	1420	0.7±0.1	0.9±0.1	0.5±0.1	0.6±0.0	0.6±0.1	0.6±0.0	0.8±0.2	0.6±0.1	0.6±0.0
15	Alpha-guiaene	1439	1435	0.1±0.1	0.3±0.2	0.1±0.0	0.1±0.0	0.0±0.0	0.2±0.1	0.1±0.1	0.3±0.0	0.1±0.1
16	Alpha-humulene	1454	1457	0.9±0.2	1.0±0.1	0.1±0.0	0.8±0.0	0.7±0.0	0.8±0.1	0.9±0.1	0.9±0.1	0.7±0.1
17	Germacrene-D	1480	1482	0.6±0.2	0.6±0.1	0.6±0.1	0.6±0.0	0.6±0.1	0.5±0.0	0.7±0.0	0.6±0.1	0.6±0.0
18	Pentadecane	1500	1500	0.6±0.1	0.7±0.1	0.6±0.1	0.6±0.1	0.7±0.0	0.6±0.1	0.7±0.1	0.5±0.2	0.5±0.0
19	Farnesene	1508	1510	0.2±0.0	0.2±0.0	0.2±0.0	0.2±0.0	0.3±0.0	0.3±0.0	0.3±0.1	0.2±0.0	0.2±0.1
	**Sub Total**			4.0±0.1	4.5±0.1	3.0±0.1	3.5±0.03	3.5±0.04	3.6±0.07	4.9±0.02	3.7±0.07	3.2±0.04
	**Aliphatic Hydrocarbon**											
20	Heptadecane	1700	1697	0.1±0.0	0.1±0.0	0.1±0.1	0.1±0.0	0.2±0.1	0.1±0.0	0.1±0.0	0.1±0.0	0.2±0.1
21	Octadecane	1800	1796	0.2±0.1	0.2±0.0	0.1±0.0	0.2±0.0	0.2±0.1	0.1±0.0	0.1±0.1	0.2±0.1	0.1±0.0
22	Nonadecene	1870	1870	3.0±1.0	2.5±0.2	1.6±0.1	3.4±0.2	3.6±2.2	2.1±0.2	2.7±0.5	2.7±0.3	2.5±0.3
23	Nonadecane	1900	1895	14.5±5.8	12.6±0.2	8.9±0.1	14.2±1.9	15.1±7.5	9.2±0.8	10.3±3.3	10.9±4.0	9.5±1.1
24	Eicosane	2000	2002	1.4±0.6	1.4±0.1	0.9±0.1	1.5±0.0	1.7±1.0	1.0±0.2	1.0±0.3	1.1±0.2	1.0±0.0
25	Heneicosane	2100	2096	6.6±3.2	6.1±0.2	3.8±0.4	7.1±0.4	8.7±5.2	4.8±1.1	4.6±1.6	5.5±1.0	4.9±0.1
26	Tricosane	2300	2298	1.3±0.8	1.3±0.2	0.7±0.0	1.7±0.2	2.3±1.4	1.1±0.3	0.8±0.5	1.3±0.2	1.1±0.1
	**Sub Total**			27.1±1.6	24.2±0.1	16.1±0.1	28.2±0.4	31.8±2.5	18.4±0.4	19.6±0.9	21.8±0.8	19.3±0.2
	**Total**			92.8±3.9	95.3±0.6	91.4±0.1	94.0±0.9	91.9±2.0	93.6±2.1	88.8±3.5	91.8±2.2	91.1±0.3

Note: WM: Without Mulch; OM: Organic Mulch; PM: Black polyethylene Mulch; ± values represents standard deviation of three replicates.

The percentage of oxygenated monoterpene and sesquiterpene components like citronellol, geranyl acetate, linalool and *trans*-rose oxide were higher in open sunny conditions as compared to 25% shade and 50% shade level (Table 4). Results were similar to the study of *S*. *sclarea* and *T*. *minuta* in which oxygenated monoterpenes and sesquiterpene components decreased with increase in shade levels [5,17]. Light played an important role in essential oils production that depends upon the metabolic processes, as well as the physiology of the whole plant. Black polyethylene mulch produced higher percentage of citronellol and *trans*-geraniol followed by organic and without mulch. This may be because of biosynthetic activation of pathways of essential oils due to light which depend on the carbon chain obtained by photosynthetic light [10]. The remaining compounds did not showed any consistent trend under shade levels and mulch types. The variation in the compounds of damask rose essential oil may be related both to the availability of light and favorable soil temperature and soil moisture, since the quality of essential oil is determined by favorable environmental conditions in the productivity of secondary metabolites of essential oil crops [36].

### 3.8 Principal components analysis

**Fig 3:**
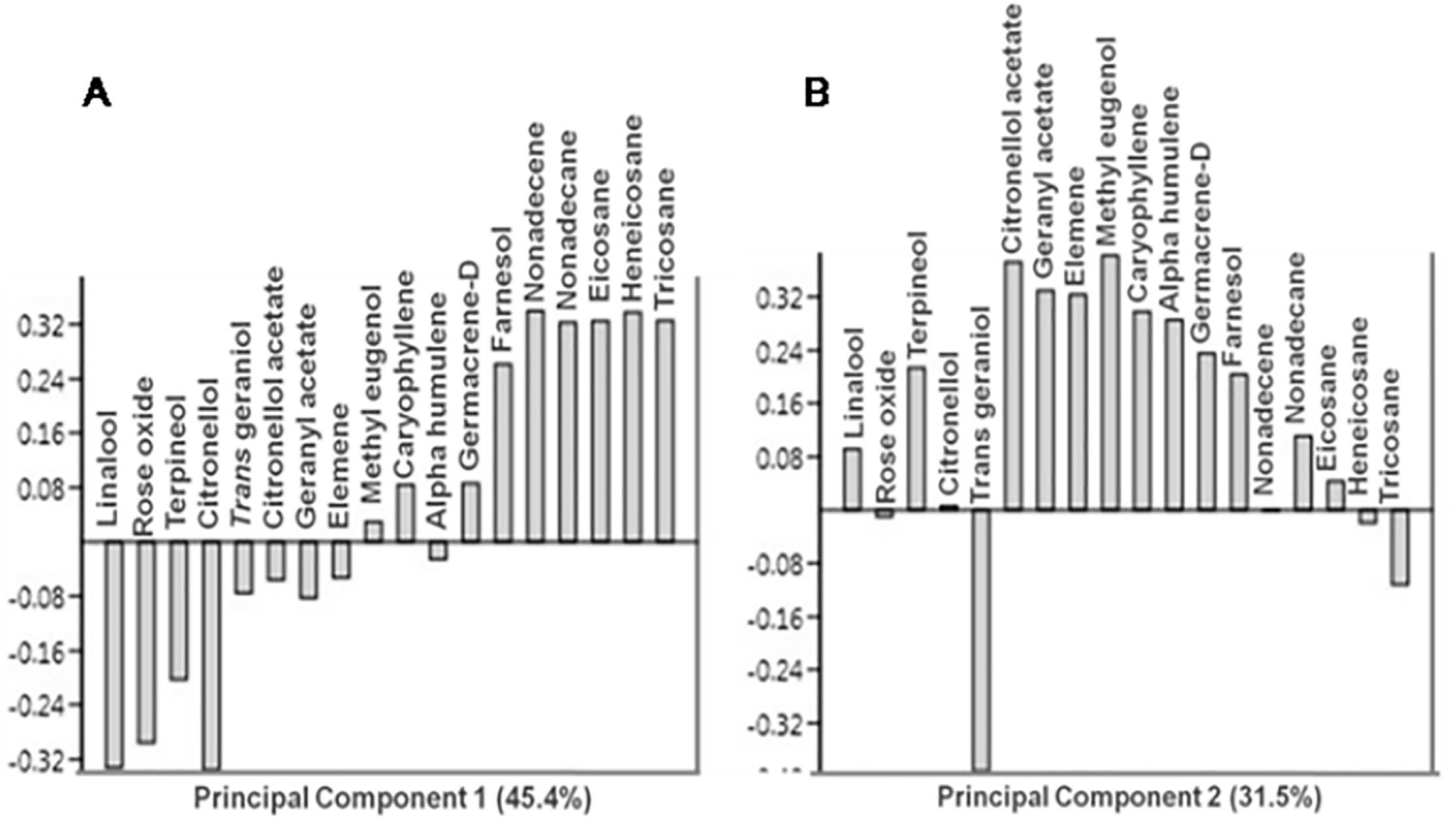
**Loading plot of principal component analysis (A) loading plot PC-1 (B) loading plot PC-2**.

Eighteen components of damask rose essential oil from different nine treatments were submitted to principal component analysis (PCA) for analyzing compositional and different treatments variation. PC-1 and PC-2 contributed to 45.4 and 31.5% of variance which jointly explained 76.9% of total variance (Fig 3). PC-1 clearly distinguishes the farnesol, nonadecane, nonadecene, eicosane, heneicosane and tricosane samples having positive loading, however, linalool, rose-oxide, terpineol and citronellol samples are negatively loaded (Fig 3A). PC-2 is mainly separated by positive loading of linalool, rose-oxide, citronellyl acetate, geranyal acetate and methyl eugenol rich samples. Negative loading is due to trans-geraniol and tricosane (Fig 3B).

**Fig 4:**
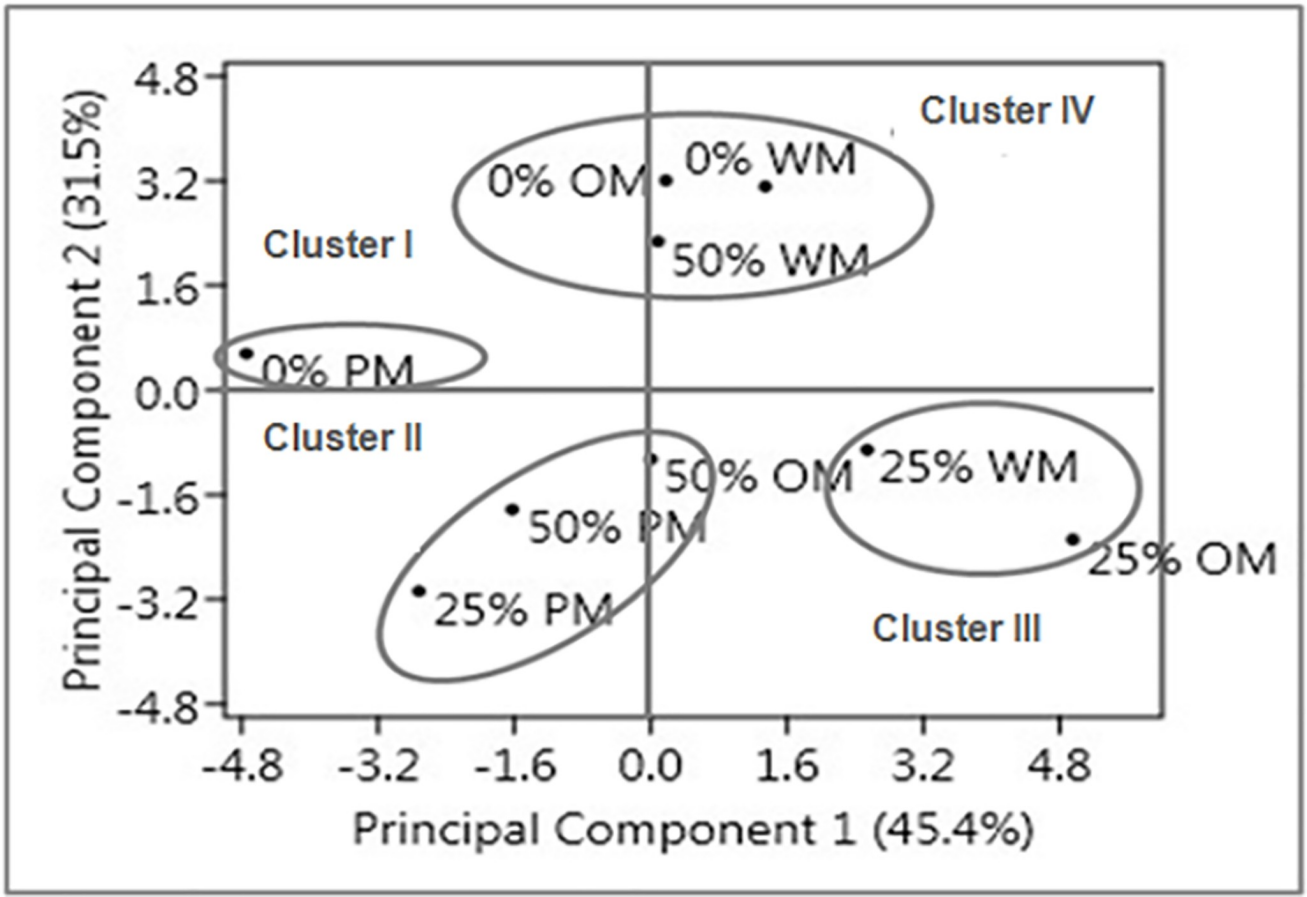
Principal component analysis of essential oil compounds data. Principal component 1 and Principal component 2 jointly explained 76.9% of the total variation. 0%, 25% and 50% are the shade levels, respectively, while WM, OM, PM are the mulch types *viz*., without mulch, organic mulch and black polyethylene mulch, respectively.

Four distinct clusters were observed in score plot (Fig 4). Cluster I (0% PM) explained higher concentration of citronellol in open sunny condition with the applications of black polyethylene mulch. In Cluster II (25% PM, 50% OM and 50% PM) concentration of citronellol, trans-geraniol and geranyl acetate was found higher in 25% shade level with the application of black polyethylene followed by 50% black polyethylene and organic mulch. Cluster III (25% WM and 25% OM) showed that under mulching conditions, higher concentration of trans-geraniol, geranyl acetate, nonadecene, nonadecane and heneicosane were observed. In cluster IV (0% WM, 0% OM and 50% WM) plants grown under open sunny conditions along with the application of mulch, produced the higher concentration of citronellol and trans-geraniol. However, percentage of these major compounds decreased under 50% shade level (Table 5). The analyzed data revealed that the components hydrocarbon *viz*., nonadecene, nonadecane, eicosane, heneicosane and tricosane are positively correlated with each others. Hence it was found that monoterpenes and hydrocarbons formed inverse relationship among themselves.

**Table 5:** Clusters variability in major essential oil constituents (%) of damask rose as affected by shade level and mulch type.

Sr. No.	Components	Cluster 1	Cluster II	Cluster III	Cluster IV
1	Citronellol	49.2	44.2–47.1	35.5–40.8	40.0–45.5
2	Trans-Geraniol	14.4	15.6–18.1	15.1–15.3	12.6–13.0
3	Geranyl acetate	2.1	1.0–1.6	1.2	1.7–2.8
4	Nonadecene	1.6	2.0–2.7	3.4–3.6	2.5–3.0
5	Nonadecane	8.9	9.0–10.9	14.2–15.1	11.3–14.5
6	Heneicosane	3.8	4.5–5.5	7.1–8.7	5.0–6.6

## 4. Conclusions

Applications of ‘‘different shade levels and mulch types” influenced the growth, yield attributes and quality of essential oil of damask rose under the mid hill conditions of Western Himalayas. The open sunny conditions and black polyethylene mulch promotes the production of major secondary metabolites of damask rose. Since the adaptation of plants to shaded conditions have reduced the yield attributes, hence from the studies we concluded that plants grown under open sunny condition with black polyethylene mulch produced higher yield and good quality of damask rose.
